# Supplementary material for: The Effects of Red Light on Mammalian Sperm Rely upon the Color of the Straw and the Medium Used
Source: Animals (Basel). 2021 Jan 8;11(1):122. doi: 10.3390/ani11010122 (PMC7826721; doi:10.3390/ani11010122)
Supplement: Supplementary file 1 [file animals-11-00122-s001.pdf]

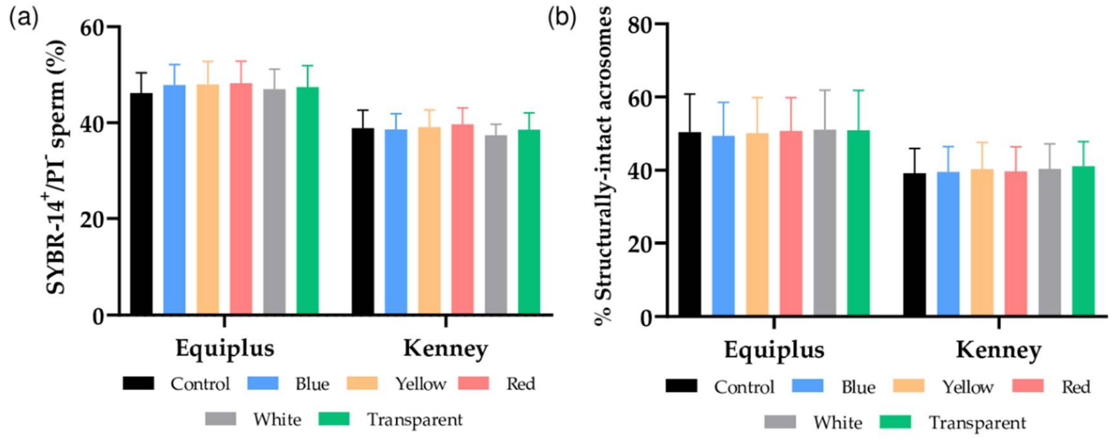

Figure S1. Effects of the color of the straw, extender and light-stimulation on the percentages of membrane-intact spermatozoa ($SYBR14^{+}/PI^{-}$; **a**) and acrosome integrity ($PNA\text{-}FITC^{-}/PI^{-}$; **b**) in control (non-irradiated) and irradiated samples packed into straws of different color and extended either with Equiplus or Kenney. No significant ($p>0.05$) differences were found between non-irradiated (control) and irradiated samples, regardless of the straw color or the extender used to dilute the semen. Data are shown as mean ± SEM of thirteen separate experiments.

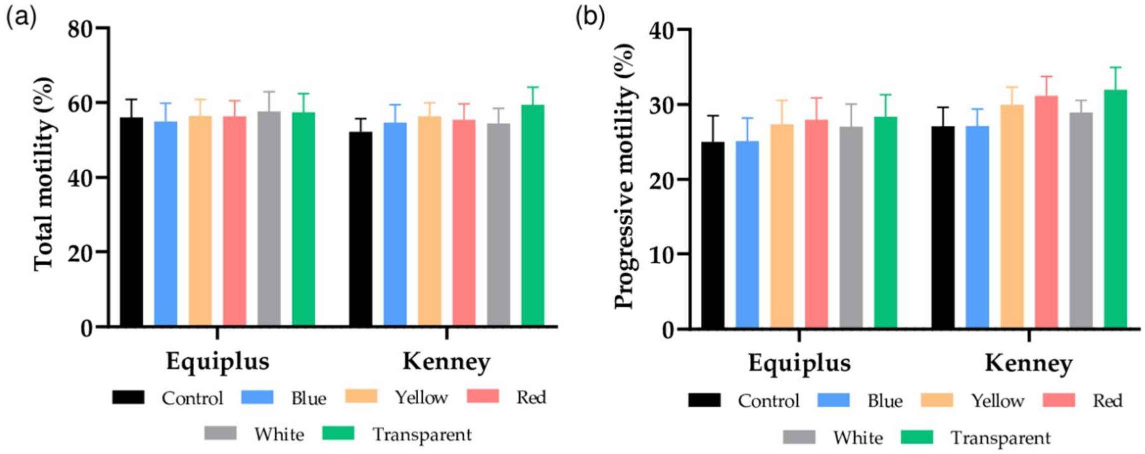

Figure S2. Effects of the color of the straw, extender and light-stimulation on the percentages of total (**a**) and progressive (**b**) motility on the control (non-irradiated) and irradiated samples packed into straws of different color and extended either with Equiplus or Kenney. No significant differences were found between non-irradiated (control) and irradiated samples, regardless of the color of the straw and the extender used. Data are shown as mean ± SEM of thirteen separate experiments.

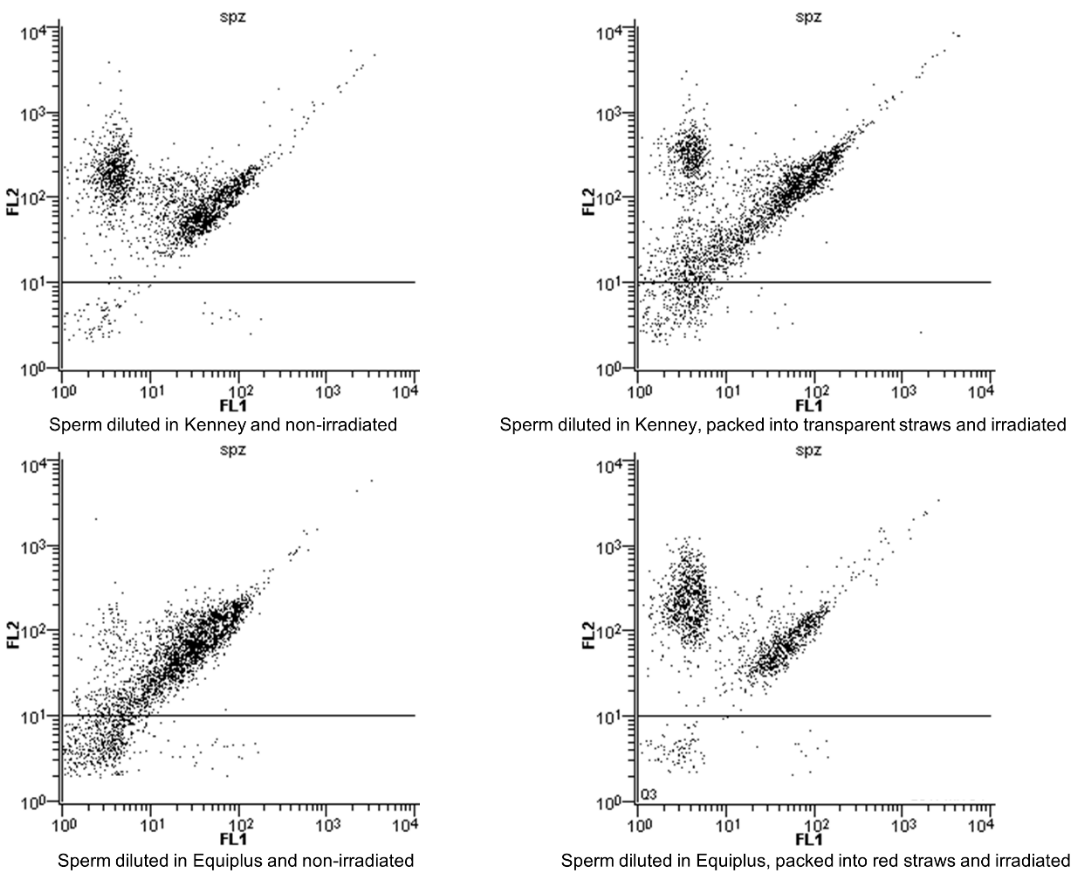

Figure S3. Representative dot-plots for JC1 staining (non-irradiated sperm diluted in either Kenney or Equiplus; sperm diluted in Kenney extender, packed into transparent straws and irradiated; and sperm diluted in Equiplus extender, packed into red straws and irradiated).

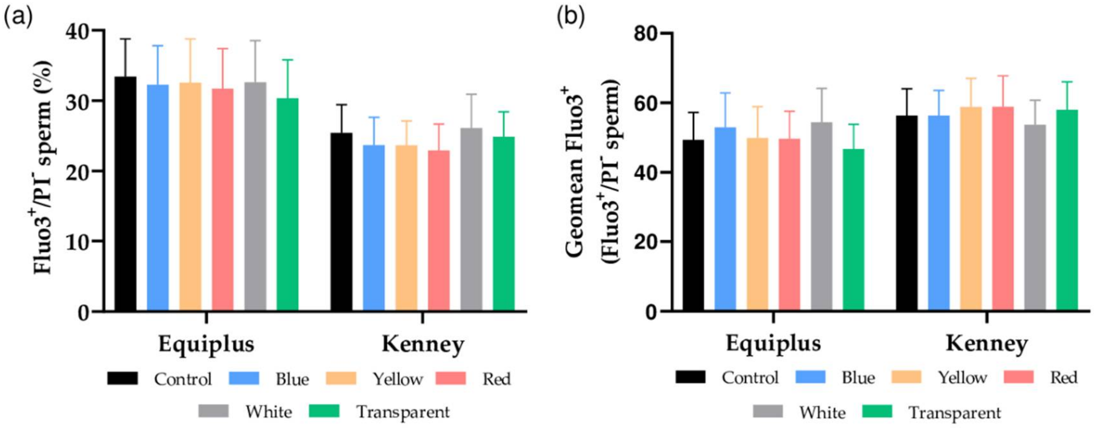

Figure S4. Effects of the color of the straw, extender and light-stimulation on intracellular calcium levels in control (non-irradiated) and irradiated samples packed into straws of different color and extended either with Equiplus or Kenney extenders. Data are shown as: (**a**) percentages of spermatozoa with high intracellular calcium levels ($Fluo3^+$) and (b) geometric mean intensity of $Fluo3^+$ in the sperm population with high intracellular calcium levels ($Fluo3^+$ sperm). No significant differences were found between non-irradiated (control) and irradiated samples, regardless of the color of the straw and the extender used. Data are shown as mean ± SEM of thirteen separate experiments.
